# Prevalence, Awareness, Treatment, and Control of Hypertension in Rural Areas of Davanagere

**DOI:** 10.4103/0970-0218.62578

**Published:** 2010-01

**Authors:** Yuvaraj BY, Nagendra Gowda MR, Umakantha AG

**Affiliations:** S.S Institute of Medical Sciences, Davanagere, India; 1 Basaveshwara Medical College and Hospital, Chitradurga, India; 2 J. J. M. Medical College, Davanagere, India

**Keywords:** Awareness, hypertension, prevalence

## Abstract

**Objective:**

To study the prevalence, awareness, treatment, and control of hypertension in the rural areas of Davanagere.

**Type of Study:**

Cross-sectional community-based study.

**Setting:**

Villages belonging to six sectors of the Davanagere Taluk.

**Materials and Methods:**

General population above 18 years.

**Methodology:**

A community-based sample was chosen by a multistage sampling technique. Subjects were screened for hypertension by a house-to-house survey. Subjects with systolic blood pressure more than 140 and diastolic blood pressure more than 90 mm of Hg, on hypertensive treatment, and history of hypertension were classified as hypertensives. The data thus obtained was compiled and analyzed.

**Results:**

The prevalence rate of hypertension in the study population was 18.3% (95% CI, 16.7-19.9%). Prevalence of hypertension was more in males 19.1% (95% CI, 16.7-21.5%) than in females 17.5% (95% CI, 14.9-20.1%); 11.6%, 5.6%, and 1.2% of the total subjects had Grade I, Grade II, and Grade III, respectively. Only 33.8% of them were aware of their hypertensive status. Hypertensives of 32.1% were on treatment, and 12.5% adequately controlled their BP. About 6.9% of the total hypertensives had severe hypertension.

**Statistical Analysis:**

Proportions, One way Analysis of Variance, Chi-square test.

## Introduction

Hypertension is an interesting disease entity of its own. It remains silent, being generally asymptomatic during its clinical course. As it is hidden beneath an outwardly asymptomatic appearance, the disease does immense harm to the body in the form of ‘Target Organ’ damage, hence, the WHO has named it the ‘Silent Killer’.(1)

The prevalence of hypertension is increasing in trend. As most of the urban areas have access to health facilities, the hidden mass of hypertension in the community can be detected and treated. However, the situation is reversed in rural areas.

A majority of the rural population in India have inadequate access to healthcare. Over half of the outpatient consultations are with indigenous and private practitioners, where regular screening for hypertension is not practiced. Clinic-based (Opportunistic) screening of hypertension will not screen and detect a large proportion of adult hypertensives. In turn they will not seek healthcare from the formal health sector, until seriously ill. Community-based screening can improve the detection and treatment of Hypertension. As fewer studies have been undertaken in rural India, it was decided to assess the prevalence, awareness, treatment, and control of hypertension particularly among the rural population of Davanagere taluk.

## Materials and Methods

A community-based cross-sectional survey was conducted in the villages of Davanagere taluk.

A multistage sampling procedure was used to select the study subjects. At the first stage, Davanagere taluk was divided into six diagonally equal sectors on a geographical map. All the Primary Health Care (PHC) units falling in the sector were listed and every second PHC from each sector was chosen by systematic random sampling. In the second stage, villages in the selected PHCs were listed and chosen by using a random number table. In the third stage, a door-to-door survey was carried out and all the members aged above 18 years, present in the household during the time of the survey, were included in the study.

An informed verbal consent was obtained before measuring the blood pressure. One thousand nine hundred subjects drawn equally from the six sectors constituted the study sample. Blood pressure was measured by auscultation, using the standardized sphygmomanometer. All the participants were requested to take rest for ten minutes. Blood pressure was measured in the sitting posture with an appropriate-sized cuff encircling the arm. Two separate readings were taken at an interval of minimum three minutes. The average of the two readings was taken. Systolic BP measured at the appearance of the Korotkov's sounds (Phase I) and Diastolic BP was taken at the point of disappearance of the sounds (Phase V). The information regarding age, sex, awareness, and treatment of hypertension was collected in a pre-designed proforma. The participants with history of hypertension and on hypertensive medication were also labeled as hypertensives. Recent JNC VII(2) and WHO(3) classification were used for classifying the hypertension.

## Results

A total of 1900 subjects residing in rural areas were screened for hypertension, of which 349 had hypertension, giving the prevalence rate of 18.3% (95% CI, 16.7 – 19.9%) [Table 1]. The prevalence of hypertension was more in males 19.1% (95% CI, 16.7 – 21.5%) than in females 17.5% (95% CI, 14.9 – 20.1%). An upward trend in prevalence was observed with increase in age, especially above 40 years, in both the sexes. Males of all the age groups had a higher prevalence than females, except the 30–39 year age group. The prevalence of hypertension was 4.9% in the 18–29 year age group, which increased to 31.2% in subjects of age over 70 years. This difference was statistically significant. The mean SBP and DBP increased gradually with increase in age. This was also statistically significant. There was no change in the mean systolic and diastolic BP in both the sexes.

**Table 1:** Distribution of screened population and hypertensives according to age and sex

Age group (in Years)	Male		Female		Total			
			
	Screened population	Prevalence (%)	Screened Population	Prevalence (%)	Total screened population	Prevalence (%)	SBP in mm Hg mean (SD)	DBP in mm Hg mean (SD)
Below 29	234	5.9	214	3.7	448	4.9	119.8 (11.1)	77 (8.0)
30–39	250	11.2	225	12.9	475	12	125 (12.5)	79.5 (8.4)
40–49	206	25.7	158	25.9	364	25.8	134.9 (15.5)	81.9 (10.3)
50–59	182	26.9	119	26.1	301	26.6	139.7 (18.6)	83.2 (10.2)
60–69	127	30.7	86	30.2	213	30.5	139.7 (20.4)	83.8 (11.7)
70 and above	54	33.3	45	28.9	99	31.3	140.8 (21.5)	83.6 (11.8)
Total	1053	19.1	847	17.5	1900	18.37	128.5 (16.97)	80.6 (9.91)
*P* value		0.36Ψ			<0.0001Ψ		< 0.0001*	< 0.0001*

Ψ Chi-square test

* One way ANOVA

The blood pressure levels of subjects, classified according to the WHO-defined grades are shown in Table 2. Overall, 11.7% of the study subjects had optimal blood pressure; a total of 15.7% of the subjects had high normal blood pressure; 11.6%, 5.6%, and 1.2% of the subjects had Grade I, Grade II, and Grade III, respectively. Higher proportions of males had Normal, Optimal, Grade I, and Grade II hypertension, but more number of females had high normal BP. However, the figures may be underestimated because some of the subjects were on antihypertensives.

**Table 2:** Distribution of blood pressure levels by WHO grades

Group	Blood pressure level	Male n (%)	Female n (%)	Total n (%)
No with normal BP	SBP < 120 mmHg and	591 (31.1)	439 (23.1)	1030 (54.2)
	DBP < 80 mmHg.			
No with optimal BP	SBP = 120 – 129 mmHg, and/or	128 (6.7)	95 (5.0)	223 (11.7)
	DBP = 80 – 84 mmHg.			
No with high normal BP	SBP = 130 – 139 mmHg or	133 (7.0)	165 (8.7)	298 (15.7)
	DBP = 85 – 89 mmHg.			
Grade 1	SBP = 140 – 159 mmHg or	131 (6.9)	90 (4.7)	221 (11.6)
	DBP = 90 – 99 mmHg;			
Grade 2	SBP = 160 – 179 mmHg or	58 (3.1)	47 (2.5)	105 (5.6)
	DBP = 100 – 109 mmHg			
Grade 3	SBP > or = 180 mmHg and	12 (0.6)	11 (0.6)	23 (1.2)
	DBP > or = 110 mm Hg.			

Among the 349 hypetensives only 33.8% were aware of their hypertensive status [Table 3], as hypertension is a disease that occurs without any symptoms, and is only manifested in the late stage. A majority of the subjects were young hypertensives, in the 18 - 39 year age group.

**Table 3:** Awareness of hypertension according to age and sex

Age group (in years)	Male			Female		
		
	Known n (%)	Unknown n (%)	Total n (%)	Known n (%)	Unknown n (%)	Total n (%)
Below 29	-	14 (4.0)	14 (4.0)	-	8 (2.3)	8 (2.3)
30–39	3 (0.9)	25 (7.2)	28 (8.0)	4 (1.1)	27 (7.2)	29 (8.3)
40–49	10 (2.9)	43 (12.3)	53 (15.2)	18 (5.2)	23 (6.6)	41 (11.8)
50–59	22 (6.3)	27 (7.7)	49 (14.0)	19 (5.4)	12 (3.4)	31 (8.8)
60–69	16 (4.6)	23 (6.6)	39 (11.2)	9 (2.6)	17 (4.9)	26 (7.5)
70 and above	8 (2.3)	10 (2.9)	18 (5.2)	9 (2.6)	4 (1.1)	13 (3.7)
Total	59 (16.9)	142 (40.7)	201 (57.6)	59 (16.9)	89 (25.5)	148 (42.4)

Table 4 displays the treatment and control of hypertension. 32.1% of the total hypertensives were on treatment and only 12.5% adequately controlled their BP. About 6.9% of the total hypertensives had severe hypertension.

**Table 4:** Treatment and control of hypertension

Age group (in years)	Aware hypertensives n (%)	Treatment n (%)	Control (Total HTNs) n (%)	Control(Treated HTNs) n (%)	Severe hypertension n (%)
Below 29	-	-	-	-	-
30–39	7 (5.9)	3 (0.9)	1 (0.9)	1/3 (33.3)	1 (0.3)
40–49	28 (23.7)	21 (6.0)	6 (5.4)	6/21 (28.6)	2 (0.6)
50–59	41 (34.7)	38 (10.9)	3 (2.7)	3/38 (7.9)	13 (3.7)
60–69	25 (21.2)	21 (6.0)	4 (3.6)	4/21 (19.0)	5 (1.4)
70 and above	17 (14.4)	14 (4.0)	-	0	3 (0.9)
Male	59 (16.9)	49 (14.0)	7 (6.3)	7/49 (14.3)	12 (3.4)
Female	59 (16.9)	48 (13.8)	7 (6.3)	7/48 (14.6)	12 (3.4)
Total	349 (100)	112 (32.1)	14 (12.5)	14/112 (12.5)	24 (6.9)

## Discussion

Hypertension is a major public health problem in India and the world. Various studies conducted across the country have estimated the prevalence of hypertension ranging from 1.99% in 1958 to 21.2% in 1994. However, the study results were not consistent, due to variations in the cut-off values and also differing age groups constituting the study population. In most developing countries hypertension is increasing in trend in both urban and rural areas. The prevalence of hypertension in this study is higher than in the other Indian studies conducted in rural areas.(4–6)

The prevalence of Grade I hypertension was more compared to the other studies. However the proportions of different grades are lower compared to a study conducted in Bangladesh and India.(7)

The awareness, treatment, and adequacy of control of hypertension in this study were low (33.8%, 32.1%, and 12.5%, respectively). In a study in Kerala, it was 39%, 29%, and 10%, respectively, in the urban elderly population.(8) In another study in Kerala, the awareness was 45% in the elderly population.(9) These results might be due to a majority of younger population residing in rural areas. However, quite similar to the studies conducted in many developing countries, many factors play an important role for low awareness of hypertension. In a study in USA, control was only in 24% of all hypetensives and 45% of the treated hypertensives.(7) In Assam about 4% of all hypertensives and 18.1% of treated controlled their blood pressure.(10)

The low awareness and treatment of hypertension in this study might be due to competing health priorities with many communicable diseases and MCH services. Availability, accessibility, and lack of private services in rural areas and the silent nature of the problem plays an important role. Other factors like lower socioeconomic status, health perception and low level of education can also be cited. The low rates of awareness, treatment and control of hypertension are a matter of concern for the already outstretched public health facilities.

## Conclusion

From this study it is evident that hypertension is not only a concern of the urban population, but also a matter of debate in rural areas. Most of the hypertensives were not aware of their blood pressure, and the treatment rate was poor followed by control of hypertension. Hypertension simulates Iceberg phenomenon of diseases. Therefore, a stringent public health effort is the remedial for the detection, control, and prevention of complications of hypertension. Basic health workers can be trained for detection of hypertension, followed by strengthening of public health surveillance. Private practitioners also have to play an important role in opportunistic screening and treatment of this disease.
